# Interoception the foundation for: mind’s sensing of ‘self,’ physiological responses, cognitive discrimination and dysregulation

**DOI:** 10.1080/19420889.2020.1846922

**Published:** 2020-12-10

**Authors:** Pollard-Wright Holly

**Affiliations:** Independent Research, NCIS, Battleboro, VT, USA

**Keywords:** Interoception, mind, energy, emotional Dysregulation, consciousness, bayesian Inference, interoceptive Exposure

## Abstract

This article presents a theory of mind whereby interoception (i.e., a sense of signals originating from the body) provides a transdisciplinary framework in which theories from diverse fields may be conformed to ideas from other areas of science. Through a science of interoception, the mind itself investigates the mind and thus can explore how the universe and consciousness came about and understand how interoceptive processing is shaped by experience. Interoception provides a metastable network that enables individuals to compute the significance of stimuli as physiological changes in its complex global context. Both sensory and much cognitive discrimination and integration are affected by the flow of interoceptive information that acts as *cues* whereby unconscious events may be correlated with conscious events and the reportable content of mental life. Heightened interoceptive sensitivity and individuals who show augmented interoceptive sensitivity are susceptible to a wide range of neuropsychiatric as well as general medical conditions. Physiological responses can be measured and interoceptive awareness cultivated to generate well-being and stress resilience in the treatment of emotional dysregulation and interoceptive abnormalities.

## Introduction

This is a theory of mind and thus a theory of everything that is the content of consciousness. This includes the origins of the universe and emergence of conscious observers that suffer. Suffering as conceptualized by this theory signifies diversity in maladies of perception with interoceptive origins. According to this theory, the mind as a single fundamental entity without beginning or end is not fully conceivable. Nevertheless, the mind can transform into other forms by virtue of its motion. In this case, the mind transforms into the *potential energies*: pure awareness, mental images, and pure mental. These are potential energies because of their relationship relative to the mind. These terms were non-dogmatically chosen to explain the activities of the mind as three forms of energy and are constructs that have varied definitions across the literature. The *law of conservation of energy* is an abstract idea [1] that this theory relates to the mind. Due to the law of conservation of energy, the mind cannot be created or destroyed but as energy can be converted from one form into another. Accordingly, the mind can exist as three forms of energies, can be transformed from one form to another, and includes the capacity to carry out tasks requiring continuous repeated activity. In general the energies pure awareness, mental images, and pure mental are change which may be without pattern or patterned in a way that can be described as oscillating. According to this theory, when the mind transforms itself into the energy pure awareness it does so forming no discernible patterns and thus change is patternless. Thereby mind (as the energy pure awareness) represents a certain continuity that exists without a characteristic or trait by which one can describe it by observation, measurement, or combination. Nevertheless, the mind as embodied energies (i.e., mental images and pure mental) generates information that is patterned. This theory contextualizes ‘reality’ as a viewpoint of consciousness by mind (as an ‘observing ego’) with the perception of being an individual. This will be discussed further by way of the text. Mind (as an ‘observing ego’) is that which defines conscious events, but which is not consciousness in and of itself [2]. Accordingly, mind (as an ‘observing ego’) may conceptualize change in a myriad of ways, for example via theoretical physics and thus ‘reality’ is created by oscillating information, oscillating strings of energy, forces, and oscillating charges. Alternatively, mind (as an ‘observing ego’) may conceptualize change by way of neurology and thus ‘reality’ is created by brains with firing neurons that generate oscillating electrical patterns. In this article, a theory of mind will be discussed that includes fundamental frameworks derived from a melding of theories from neurology, computer science, and psychology. It is hoped that this integrative effort will advance the understanding of the mind and how interoception underlies the whole *mind-body-consciousness* setup. To this end, the author introduces a model, which describes the mind structured as energies (i.e., pure awareness, pure mental and mental images). The advantages of this theory are then discussed arguing that mind as embodied energies (i.e., mental images and pure mental) may be understood through a reinterpretation of existing theories. This is followed by contextualizing primordial feelings and Feelings of Knowing (FOKs) as representing oscillations of change and thus building blocks for cognitive context that includes interoceptive processes. A reinterpretation of the *interoceptive network* is also discussed, arguing that this represents a simple transdisciplinary framework whereby participants working in diverse fields can use to dialog. The author then describes how mind may function as embodied energies (i.e, pure mental and mental images) and (an ‘observing ego’) through an emerging predictive coding model and thus a reinterpretation of active (Bayesian) inference [3]. The key role of interoception is then conceptualized by way of a scheme whereby mind as embodied energies (i.e., pure mental and mental images) and (an ‘observing ego’) represents an organization composed of many modules that process incoming information and has an output in the form of intelligent behavior. This relationship is depicted by way of a components map model. Finally, the author concludes with theoretical issues, namely, the concept that maladaptive construal of bodily sensations [4] may lie at the heart of many neuropsychiatric as well as general medical conditions. This argues that interoceptive exposure (IE) can be used therapeutically to restore well-being and stress resilience in the treatment of emotional dysregulation and interoceptive abnormalities.

## The mind structured as energy

**Figure 1.**
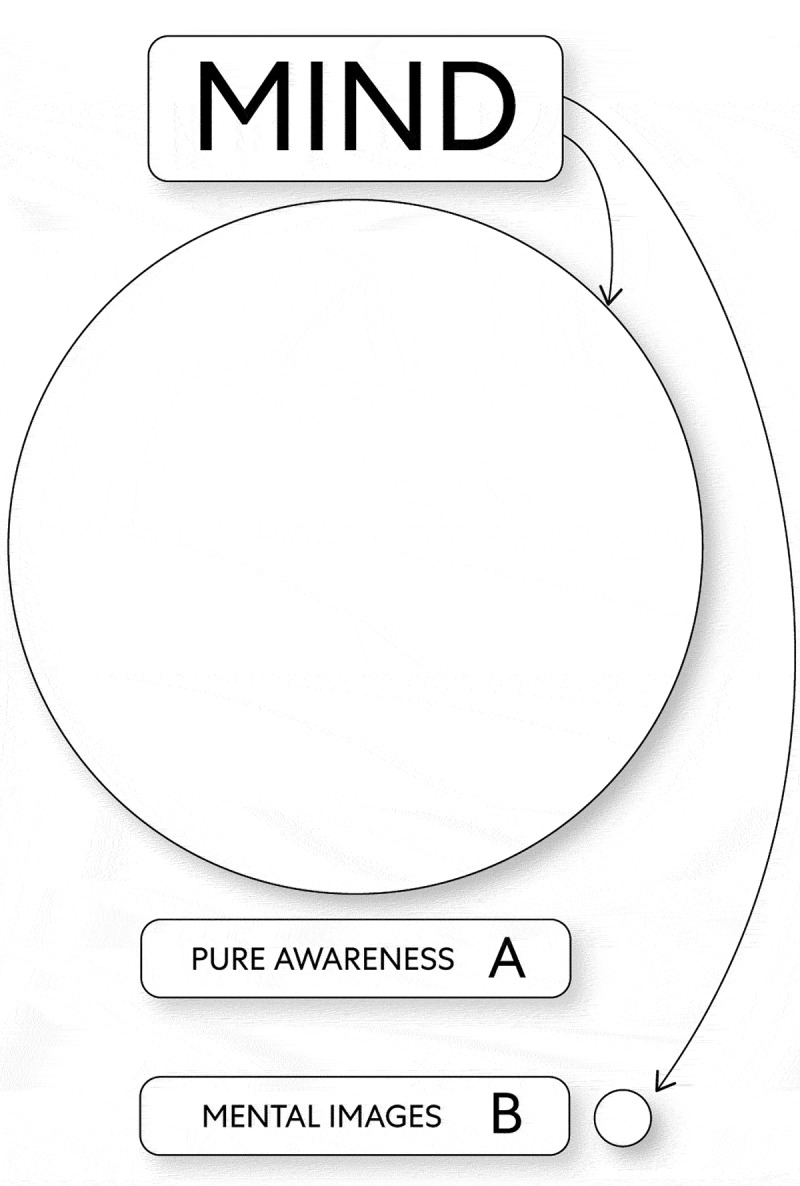
Mind Transforms Itself Into Energy

The basic premise of this theory is that mind (as the energy pure awareness) acts as the substrate for the mind’s innumerable embodied states as two energies (i.e., mental images, and pure mental) in unique cause and effect relationships. According to this theory, the universe’s origin centers on events when mind as a fundamental entity without beginning or end simultaneously transformed itself into two different forms of energy: the energy mental images and the predominant energy pure awareness [5]. Accordingly, the mind transformed itself into energy and thus the ability to act in a particular way. However because mind was in a much smaller form as the energy mental images, than it was as the energy pure awareness, this prevented it from creating meaningful relationship with itself (see, Figure 1).

A. The energy pure awareness B. The energy mental images

**Figure 2.**
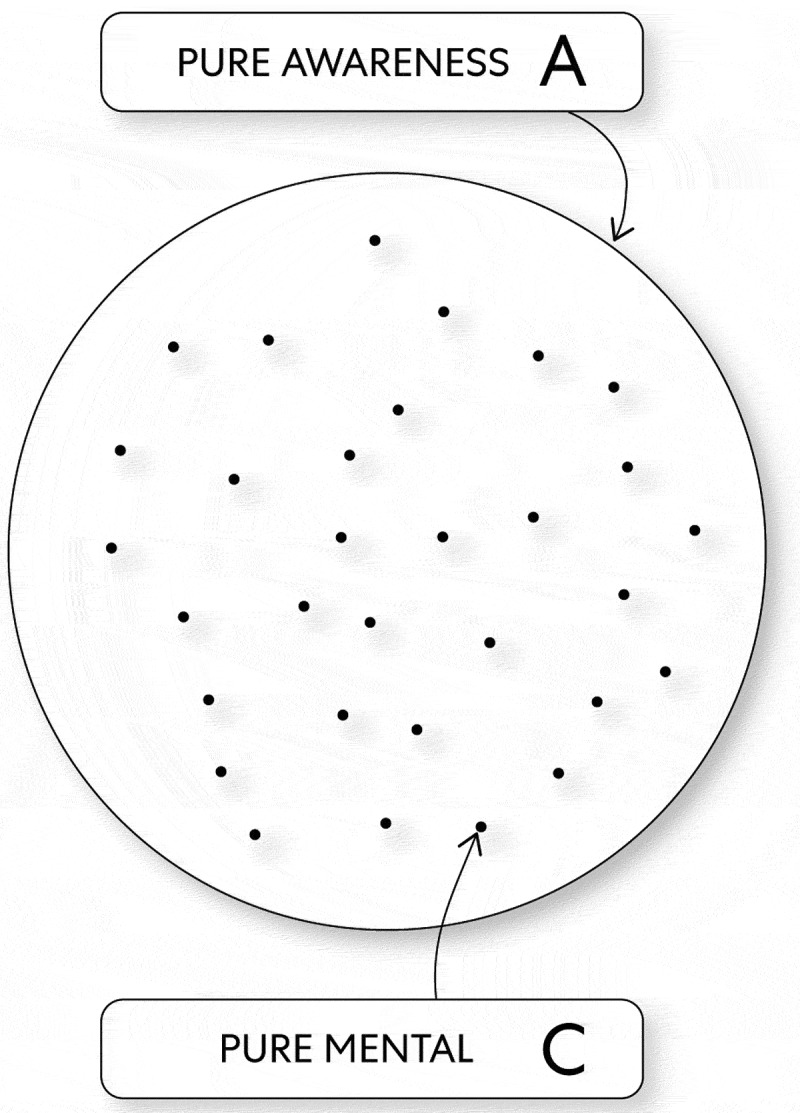
Cosmic Miraculous Conception

Therefore, mind (as the energy pure awareness) moves in a manner by which motion occurs at intervals and in this way creates oscillations of change as contractions. Thereby mind (as the energy pure awareness) transforms into the energy pure mental as innumerable focal points and thus can create innumerable-embodied states (see, Figure 2).

A. The energy pure awareness C. The energy pure mental

**Figure 3.**
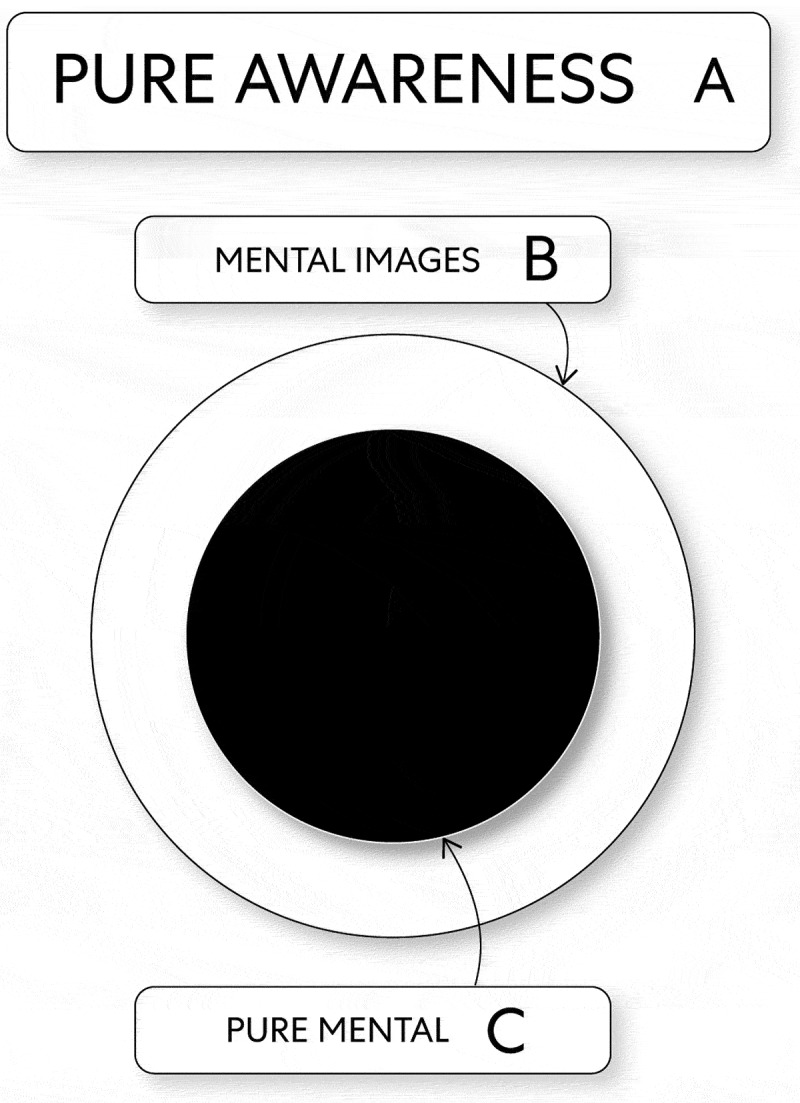
The Structure of Mind as Energy

To begin with, mind (as the energy mental images) moves in a regular manner and thus creates a definite pattern. By doing this, it moves toward itself as focal points of the energy pure mental. Accordingly, by way of oscillations of change, mind creates relationships with itself as two energies (i.e., mental images and pure mental). In this way, focal points of the energy pure mental are contained by the energy mental images (see, Figure 3).

B. The energy mental images embodying the energy pure mental C. The energy pure mental embodied by the energy mental images

**Figure 4.**
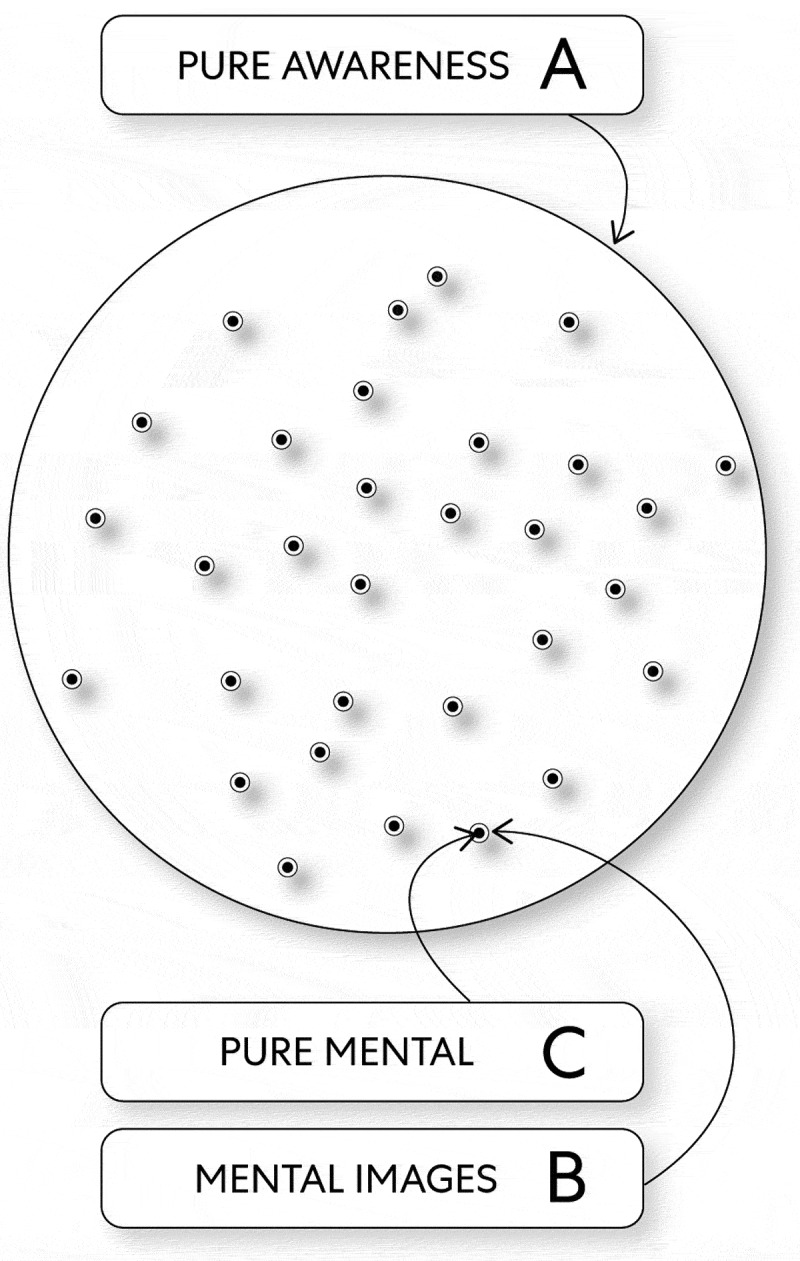
The Structure of Mind as Energy

Therefore, mind ‘embodies’ itself and thus creates innumerable relationships by way of structure:

1. ‘Body’: the energy pure awareness creates a substrate for embodied relationships.
2. Framework: the energy mental images oscillate and thus produce change that will be encoded to create the content for ‘consciousness.’
3. ‘Mind’: the energy pure mental engages in unconscious activities (i.e., events) that are necessary to create ‘consciousness’ (see, Figure 4).

A. The energy pure awareness B. The energy mental images C. The energy pure mental

Mind (as the energy mental images) by way of actions and reactions oscillates, thus changes. Mind (as the energy pure mental) by way of actions and reactions can *detect, process*, and *promote* different modes of oscillation, thus changes and keeps a record of this [6]. Accordingly, actions and reactions are stored by mind (as focal points of the energy pure mental) as ‘oscillating records’ that act as ‘seeds’ that will eventually bear fruit by way of embodiment [6]. Mind (as focal points of the energy pure mental), by way of the record it keeps, calls itself into unique cyclic embodied relationships with itself. By guiding itself (as the energy mental images) to create definite patterns of oscillations of change, mind moves toward itself (as focal points of the energy pure mental).

Mind existing as two embodied energies (i.e., pure mental and mental images) interacts by way of actions and reactions. Because mind (as an ‘observing ego’) perceives this, three types of intelligence are created (to varying degrees):

1. The intelligence of conscious events: change or information created by mind (as the energy mental images) that moves in a manner that varies and thus oscillates.
2. Unconscious intelligence: primordial consciousness, proto-consciousness, or unconscious events created by mind (as the energy pure mental) that detects, processes and promotes oscillations and thus change (i.e., information).
3. Conscious intelligence: consciousness that is created by way of actions and reactions of mind (as the energies mental images and pure mental) in an embodied relationship and experientially by mind (as an ‘observing ego’). However, if mind as the energy pure awareness had not given ‘birth’ to itself as focal points of the energy pure mental, there would be no embodiments. Accordingly, mind as the energy pure awareness is the substantial cause of conscious intelligence consciousness.

According to this theory, mind (as the energy pure mental) functions as a detector and integrator of information [7] that is generated by mind (as the energy mental images). Mind (as the ‘observing ego’) is a unique viewpoint of consciousness that emerges due to the activities of mind (as the energy pure mental). In the first place, mind (as the energy mental images) generates an unexpected stimulus and thus sets up a processed signal to mind (as the energy pure mental) that initiates a focus of synchronized oscillations of change therein [7]. Mind (as the energy pure mental) functions as a synchrony detector that detects and reacts to the degree of synchronized oscillations of change. It responds more robustly to synchronized oscillations as bursts of change rather then to random spikes and tends to reproduce in its outputs the pattern of synchronization contained in its inputs. Accordingly, mind (as the energy pure mental) processes inputs by means of multiple competitive synchronized oscillations of change that occur at different frequencies. It thus reproduces in a small volume the activity that promotes the development of multiple, ﬂuctuating, and differential synchronous oscillations [7]. Mind (as the energy pure mental) is thus not concerned with the informational content of the oscillations of change fed into it by mind (as the energy mental images), only with the degree of synchrony. It then modulates the synchronized change as outputs that it distributes as executive signals based on this integration to mind (as the energy mental images) [7].

The unconscious activity of mind (as the energy pure mental) is conveyed via encoded signals to mind (as an ‘observing ego’). These encoded signals then act as a *Bottom-up mechanism* (described later in text) whereby mind (as ‘an observing ego’) once alerted begins to *cognitively* encode the incoming signals. In this way, one function of mind (as the energy pure mental) is related to salience processing [8]. Accordingly, unconscious activity and oscillating change as encoded signals are spontaneously transformed into primordial feelings (i.e., pleasant, unpleasant, and neutral) and Feelings of Knowing (FOKs) by mind (as an ‘observing ego’). It does this before *cognitively encoding* for interoceptive stimulus that acts as a foundation whereby change as primordial feelings and FOKs are processed and propagated into cognition. By this mechanism, mind (as the energy pure mental) plays a strong role in the interactive processes of mind (as the energy mental images), and the voluntary behavior of mind (as an ‘observing ego’). During multicenter perceptual and cognitive operations, reverberating oscillations of energy loops potentiate weak synchronizations of change due to strong synchronization activities carried out by mind (as the energy pure mental). These loops may include the correlates of consciousness that also occur without a salient stimulus [9].

## The advantages of this theory

The advantages of this theory are:

1. Its structure does not need any complex mechanisms. It represents a simple transdisciplinary framework whereby participants working in diverse fields can use to dialog. Accordingly, this theory may be applied to the history of science, cognitive science, the sociology of science, the psychology of perception, semiotics, logic, and neuroscience. This includes those fields of research occupied with aspects of knowledge (e.g., cosmology, physics, neurology, psychology, philosophy, computer science, and contemplative practice) simply by reinterpreting concepts; some examples:

### Concept reinterpretation

Pure awareness is reinterpreted as representing dark energy, substrate, or ‘body.’

Pure mental is reinterpreted as representing dark matter, ‘mind,’ claustrum, or primordial/proto-consciousness.

Mental images is reinterpreted as representing normal matter/normal energy, brain and nonclaustral structures.

Energy is reinterpreted as representing mind in a particular form, change, or information.

The dynamics of mind as embodied energies reinterpreted as representing optimization algorithms, or 'Bayesian brain.'

1. It includes the relative aspects of ‘reality’ that are the content of consciousness whereby things seem to happen in the world of appearances. The ‘absolute’ dimensions of the mind are not accessible by way of thought. However, because mind (as the energy pure awareness) links embodied states, illusions of a solid reality (‘self’ and ‘others’) emerge as the content of consciousness in a myriad of ways. This is relative truth that is only possible because cause-and-effect have no intrinsic existence [10].
2. It provides logical explanations rather than explanations that include *ex nihilo (*without a cause) [10]. This includes an explanation for interoception acting as the foundation for a sense of embodiment, motivation, and wellbeing [4] as well as emotional dysregulation and interoceptive abnormalities. Thereby it is logical to target interoception in the treatment for a wide range of mental conditions.
3. It engenders a reconceptualization of intelligence and thus ethics as relates to the treatment of non-human species. The relative aspects of ‘reality’ always link to the ‘absolute’ dimensions of the mind. The vast majority of living organisms in the world of appearances (encompassing some 99.5% of all the biomass on the earth) are non-neuronal [11] yet by way of their ‘foraging behavior’ can solve complex problems.
4. It presents a scheme for classifying Mind:
  The mind: fundamental entity with no beginning or end.
  Mind (as pure awareness): acts as the body (i.e., substrate) for all embodied states. Accordingly, it is that which grants mind (as an ‘observing ego’) the ability to instantaneously infer the presence of mind as energies (i.e., pure mental and mental images) in ‘other’ embodied states.
  Mental images: it is a form of energy that mind can transform into thus represents change that oscillates.
  Pure mental: it is a form of energy that mind can transform into and can detect, process, and promote different modes of oscillation, thus change. It can keep an ‘oscillating record’ of this [6].
  ‘Observing ego’: a viewpoint of consciousness with the perception of being an individual. It is that which defines conscious events, but which is not consciousness in and of itself [2]. Mind (as an ‘observing ego’) can sense ‘others’ presence by way of inferring pleasant, unpleasant, or neutral interoception.
5. This theory represents different approaches to the phenomenon of information and thus explicates its nature and essence by way of: mind (as the energy pure awareness), mind as embodied energies (i.e., pure mental and mental images), and mind (as an ‘observing ego’). In this context, information is energy and thus treated as essences of mind embodied. This theory melds well with Gregory Bateson’s description of information as “difference which makes a difference” and the perspective promoted by Jaime F. Cárdenas-García [12]. Accordingly, all biological information is self-produced and info-autopoiesis is a sensory commensurable, self-referential feedback process [12].

## Feeling interoception and the interoceptive network

When oscillations of change are not detected unconsciously by mind (as the energy pure mental) but instead are experienced as primordial feelings by mind (as an ‘observing ego’), this means a spontaneous transformation has taken place. Accordingly, primordial feelings act as stimuli that cause mind (as an ‘observing ego’) to interpret and process change. Thereby, it begins to think subjectively and stores change as Feelings of Knowing (FOKs). Mind (as an ‘observing ego’) experiences change as primordial feelings and reflexively discerns them as being pleasant, unpleasant, or neutral. The more intense the primordial feeling, the greater the likelihood that mind (as an ‘observing ego’) will notice it and thus create in consciousness novelty and salience [13]. When a primordial feeling (i.e., pleasant, unpleasant, or neutral) is experienced by mind (as an ‘observing ego’) it will spontaneously remember it as a Feeling of Knowing (FOK). While perceiving conscious content, mind (as an ‘observing ego’) will experience FOKs as vague judgments, intuitions, abstract concepts, and vivid expectations [2]. This creates subjectivity that is certainty but with little descriptive detail as feelings of familiarity, feelings of rightness and wrongness, or feelings of beauty and goodness [2]. Accordingly, FOKs do not fade even with ‘disuse’ nor do they fade over time; rather access to them does [5]. Therefore, by way of competing, oscillations of change as FOKs compete for the attention of mind (as an ‘observing ego’).

Mind (as an ‘observing ego’) learns experientially and by way of attention can react to primordial feelings (i.e., pleasant, unpleasant, or neutral) and FOKs. Thereby, it can alter its behavior. Accordingly, mind (as an ‘observing ego’) begins to mentally process information by reflexively reacting, and thus without volition, , to the input it receives from mind (as the energy pure mental). These reflexive reactions by mind (as an ‘observing ego’) then causes it to spontaneously create its ‘primordial sense of self’ as interoception. This sense of the internal state of the body acts as foundation for its perceiving 'real-world cognition' by way of *experience* and *observation*. Interoception is critical for a sense of embodiment, motivation, and wellbeing [4]. Accordingly, mind (as an ‘observing ego’) by way of perceiving interoception can conceptualize what it is doing as the energies mental images and pure mental. It does this by sensing FOKs (i.e., pleasant, unpleasant, or neutral) and perceiving them as interoception that becomes a powerful motivator [14,15]. What mind (as an ‘observing ego’) is actually perceiving is how busy it is (as the energy pure mental).

To explain this, first, consider that when mind is embodied as the energies mental images and pure mental it acts as a small organization. Accordingly, this organization is composed of:

1. Mind as two forms of embodied energies (i.e., mental images and pure mental) that act and react to each other.
2. Mind (as the energy pure awareness) that acts as the substrate (i.e., 'body') for embodied states.

There is an inextricable linkage between all the the unique consciousnesses created by the mind as embodied energies (i.e., pure mental and mental images). This link exists because mind (as the energy pure awareness) acts as the substrate for them. The linkage represents that which creates the viewpoint of the *holistic mind*. It is a viewpoint that is infinitely interconnected but only by reference to the ‘whole.’ This ‘whole’ refers back to the abstract idea (Feynman, 1970) of *conservation of energy*. Consciousness does not contain the mind and mind is not created or destroyed. However, mind as energy can transform from one form of energy into another. Mind (as the energy pure awareness) ‘orchestrates’ the merging of a pair or a group of embodied relationships between itself as the energies (i.e., mental images and pure mental). Therefore, communication between them is naturally simultaneous and such that one embodied relationship *infers* the presence of the other [16]. This allows mind in one embodied relationship to gain access to another by way of consciousness. When the access is authorized or unauthorized, it engages in what might be construed as the act of *mental hacking.*

When mind (as an ‘observing ego’) senses interoception it can then conceptualize itself as an organism (rather than an organization). Nevertheless, mind (as an ‘observing ego’) with the perception of being an individual often believes itself to be a living being. The crux of the difficulty lies in the fact that the perception of mind (as an ‘observing ego’) and what it ‘thinks’ it is perceiving often do not correspond [17]. Primordial feelings and FOKs are experienced by mind (as an ‘observing ego’) with the perception of being an individual and used to create sense impressions and mental events whereby neither is a ‘self’ [18]. These sense impressions and mental events are impermanent and thus do not contain or constitute any lasting separate entity that could be called a ‘self’ [18]. Accordingly, the relationship between mind as two embodied energies (i.e., mental images and pure mental) is represented both by the content of consciousness and by way of a living being (e.g., invertebrate, mammal, bird, amphibian, reptile, or fish). These living beings are *animate* ‘objects’ of consciousness that act as ‘visual’ indicators of unique embodied relationships. Thereby mind (as an ‘observing ego’) may discern the unique behavior of itself as two energies (i.e., mental images and pure mental) by way of an integrated ‘object’ (i.e., a living being). There are also *inanimate* ‘objects’ that emerge in consciousness as *living entities* (e.g., plants). These also act as ‘visual’ indicators but represent relationships between mind as three energies (i.e., pure awareness, mental images, and pure mental) that are not in an embodied relationship. Mind (as an ‘observing’ ego) is not represented by an ‘object’: it is rather represented by a unique viewpoint of consciousness. It is with this viewpoint that mind (as an ‘observing ego’) personalizes change by way of feeling it as primordial feelings and FOKs. Mind (as an ‘observing ego’) with the perception of being an individual will observe consciousness that is represented by a character (i.e., a living being). Accordingly, it will observe different events that occur in the life of this character and thus with the perception of being an individual observe, birth, old age, and death.

Mind (as an ‘observing ego’) learns experientially by observing ‘other’ unique consciousnesses and thus mind in embodied relationships (as the energy mental images and pure mental) creates distinctive characteristics. This is represented in consciousness by the emergence of innumerable species of different living beings. Empathy represents a circumstance that is only possible due to mind (as the energy pure awareness) that acts as the 'body' (i.e., substrate) for embodied relationships. Accordingly, because mind (as the energy pure awareness) creates a substrate for embodied relationships, there exists an *interoceptive network* [19]. Because the interoceptive network exists, mind can generate an awareness of itself in other embodiments. This, along with the degree of flexibility as relates to its reactions to primordial feelings and FOKs, allows mind (as an ‘observing ego’) by way of consciousness to mirror ‘another’s’ feelings [19]. Empathy refers to the ability of mind (as an ‘observing ego’) to share another ‘individual’s’ emotional states and to infer that ‘individual’s’ experiential states [19]. This has the potential to result in mind’s most genuine and effective expression of empathy toward itself.

## Mind and the predictive coding model of interoception

Mind (as the energy pure mental) may be understood to be a statistical entity of sorts that actively generates explanations for the stimuli it encounters. However, its ‘explanations,” hypothesis,’ and ‘beliefs’ are not consciously held mental states, but encoded probability distributions over the hidden causes of sensory signals [3]. Nevertheless, it is mind (as the energy pure mental) when engaged in unconscious activity consisting of detecting, binding, processing, and propagating oscillations of change, that makes the viewpoint of mind (as an ‘observing ego’) possible. Accordingly, there is a bidirectional relationship the mind has with itself as the energy pure mental and itself as an ‘observing ego’. The actions and reactions of mind (as the energy pure mental) ‘bend back on’ and thus affect an overall self-reflective process of mind (as an ‘observing ego’). This includes its perception of excitatory and inhibitory responses and in this way, it will promote or prevent an action, respectively, [20]. Mind (as an ‘observing ego’) perceives oscillations of change coherently by experiencing them as primordial feelings (i.e., pleasant, unpleasant, or neutral) and remembering them as FOKs. These feelings act as stimuli and this causes mind (as an ‘observing ego) to interpret and process change. It begins this process reflexively and in a way that creates interoception (i.e., its sense of the body ‘from within’). Interoception thereby becomes its foundation for embodied ‘selfhood’ that is a powerful set of concepts within that allows mind (as an ‘observing ego’) to conceive bodily states [3]. It regulates bodily states as *interoceptive inference* [3] and thus through attention and autonomic reflexes. In doing this, mind (as an ‘observing ego’) can integrate signals of oscillating change as primordial feelings and FOKs for cognition that may include emotion.

The attention of mind (as an ‘observing ego’) plays a prominent role in binding [21] oscillations of change that it receives and mentally processes as sensory input. In this scheme, the reactions of mind (as an ‘observing ego’) to interoception may act as a ‘searchlight’ of attention [22]. When it receives incoming oscillations of change as input from mind (as the energy pure mental) there are two immediate tasks mind (as an ‘observing ego’) must perform: (1) determine whether the input it is receiving matches its expectation of what that information should be (2) determine if the stimulus signals a state of affairs that is potentially threatening or rewarding [7]. The basic idea is that oscillations of change sent by mind (as the energy pure mental) encode the expectations of mind (as an ‘observing ego’) about the causes of sensory input [3]. In this way, the ‘current concerns’ of mind (as an ‘observing ego’) are mostly unconsciously driven [2]. When mind (as an ‘observing ego’) receives oscillations of change it experiences this as primordial feelings (i.e., pleasant, unpleasant, neutral) and FOKs interoception that acts as goal directing stimuli. *Prediction error* is the difference between:

1. encoded signals received by mind (as an ‘observing ego’) that it mentally processes as expectations of sensory input [3].
2. comparison of that input by mind (as an ‘observing ego) with predictions as FOKs interoception.

The attention and reactions of mind (as an ‘observing ego’) are how it makes interoceptive predictions across a hierarchy of perceptual processing [3]. There are fundamentally two mechanisms that allow it to do this:

1. Top-down mechanism: occurs due to the attention and reactions of mind (as an ‘observing ego’) that create FOKs interoceptive predictions. These then compete with bottom-up encoded input.
2. Bottom-up mechanism: occurs when oscillations of encoded change are sent by mind (as the energy pure mental) to mind (as an ‘observing ego’) and thus encodes its experiencing of sensory input [3].

To ensure the predictions by mind (as an ‘observing ego’) are constrained by sensory information, every top-down prediction is reciprocated with a bottom-up prediction error [3]. This mechanism ensures that the incoming oscillating change as sensory signals received by mind (as an ‘observing ego’) continuously interact with its higher-order cognitive representations. In this way, these interactions create a sense of self with motivational context [23] as goals, history, and environment and thus inform as emotional experience and motivating regulatory behavior [4,15]. The attention and reactions of mind (as an ‘observing ego’) in an embodied framework then acts as *active inference* [3]. Accordingly, mind (as an ‘observing ego’) by way of attention and reactions resolves sensory prediction errors about the state of the body in two key ways:

1. it updates its predictions as FOKs interoception to make them more like the encoded expectations [3] and thus experiences sensory input ‘as is.’
2. it resolves prediction errors, and encoded expectations become more like its predictions [3] as FOKs interoception and thus it experiences sensory input differently.

Correspondingly there is a strong consistency constraint as relates to the conscious content perceived by mind (as an ‘observing ego’). Where there are two inconsistent contents, only one can become conscious at a time; this may also apply to beliefs, as in the case of cognitive dissonance [2]. Furthermore, when mind (as an ‘observing ego’) receives redundant encoded input signals its conscious content fades, and thus ‘informativeness’ [2] seems a necessary condition for conscious perception. The spontaneous flow of thoughts is an interplay between mind (as the energy pure mental) and mind (as an ‘observing ego’). Accordingly, the world and body perceived by mind (as an ‘observing ego’) emerge from predictions [3] that involve multiple “threads” of conscious and unconscious elements [2]. The actions and perception by mind (as an ‘observing ego’) minimize the same prediction error [24]. Thereby its perception of body states (i.e., interoception) and cognitive appraisals of these states inform as response selection [4. In this scheme, the perception of mind (as an ‘observing ego’] resolves (exteroceptive) prediction errors by selecting predictions that best explain its sensations [3]. In this way, the behavior of mind (as an ‘observing ego’) suppresses (proprioceptive) prediction error by changing (proprioceptive) sensations [3]. This suppression rests on proprioceptive predictions which are fulfilled by mind (as an ‘observing ego) by way of its attention and response as reflexes. This activity then acts as input to mind (as the energy pure mental) that takes action based on the degree of oscillating change it receives. It then generates reverberating oscillations of energy loops so that weak synchronizations of change may be potentiated. Accordingly, oscillations of change are generated at the same frequency that mind (as the energy pure mental) then distributes as executive signals to mind (as the energy mental images) [7]. In this way, mind as embodied energies (i.e., pure mental and mental images) can function as an organization with a CEO/president as the intelligence of mind (as an ‘observing ego’). The attention and reactions of mind (as an ‘observing ego’) to its embodied experience is significant for its capacity for self-representation and well-being [4]. However, an over-dependence on the Top-down mechanism that manifests as conceptual (in contrast to sensory) awareness [4] may significantly limit its ability to relate to itself in other embodiments by way of the interoceptive network, and when mental hacking (as previously described).

## A components map model of mind

Mind as embodied energies (i.e., pure mental and mental images) can be conceptualized as being an organization composed of modules that process incoming information and have an output in the form of intelligent behavior [7]. Accordingly, mind as energies (i.e., pure mental and mental images) represents unconscious modules that are busy processing their own input and providing an output. Whereas mind (as an ‘observing ego’) represents the conscious module that needs to know as a whole what it is doing as energies (i.e., pure mental and mental images). This is so it knows what best to do next; otherwise, intercommunication between the modules will lead to overload [7]. When mind (as an ‘observing ego’) receives input from mind (as the energy pure mental) it will react to it (as previously described) and thus create its foundation for processing as interoception. This foundation of interoception gives mind (as an ‘observing ego’) the capacity to create its own internal map through its attention and reactions to interoceptive signals. Thereby it can review its whole embodied situation “at a glance” and thus make instant decisions [7]. Accordingly, mind (as an ‘observing ego’) not only “binds” sensory information through its attention but by way of its reactions sends back orders and calls in further reports and analyses (when needed) [7] to mind (as the energy pure mental). In this context, interoception plays a key role in structuring the experiences of mind (as an ‘observing ego’) that includes its ‘embodied selfhood’ (i.e., ‘being and having a body’) [3]. Although this ‘selfhood’ is experienced by mind (as an ‘observing ego’) as being continuous and integrated, these phenomena unfold across many partially independent and overlapping levels of description and experiences [3]:

1. being and having a body
2. perceiving the world from a first person perspective
3. intention and agency
4. being a continuous self over time
5. a ‘narrative’ self or ‘I’ that depends on episodic autobiographical memory
6. a social self shaped by being ‘me’ and by the perception of ‘others.’

Mind (as an ‘observing ego’) with a foundation of interoception uses primordial feelings (i.e., pleasant, unpleasant, and neutral) and FOKs to create (to varying degrees based on embodiment):

### Non-conceptual thoughts

1. exteroceptive body (e.g., sight, hearing, touch, smell, taste, thermoception, pain) [2]
2. proprioceptive senses (e.g., position, motion state) [2]

#### Affective states

- refers to felt states that are consciously experienced as pleasant or unpleasant, positive or negative (i.e., valenced) [25] and thus are constructs found in humans and animals alike [26]. The reactions by mind (as an ‘observing ego’) to primordial feelings and FOKs (rather than oscillations of change) conceptualized by this theory create patterns of feelings and thus affective states (i.e., felt states). In this way, faster onset and shorter duration of affective states are associated with emotion and a slower-onset, longer duration of affective states are associated with moods [25].

1. Examples of affective feelings (e.g., nociception, disgust, empathy) [27])

#### Emotions or emotion-like states

- in its broadest sense are states characterized by loosely coordinated changes in the following five areas [25]:

1. Feeling: changes in subjective experience
2. cognition: changes in attentional, perceptual, and inferential processes (appraisals)
3. action: changes in the predisposition for or execution of specific responses
4. expression: changes in facial, vocal and, postural appearance
5. physiology: changes in physiological and neural activity.
  Examples of emotions (e.g., happiness, sadness, fear, anger, surprise, embarrassment, jealousy, guilt, pride) [28]

#### Conceptual thoughts and memories

[2]:

1. perceptual stimuli (e.g., inner speech, dreams, visual imagery)
2. fleeting present and its fading traces in immediate memory
3. autobiographical episodes (experienced and recalled)
4. expectations and effortful voluntary control
5. explicit beliefs (about ‘self’; about the world)
6. novel skills, abstract concepts

Reactions are the predictor of change in mind’s relationship with itself as embodied energies (i.e., pure mental and mental images) and (as an ‘observing ego’). Accordingly, impulsive reactions and reactions with forethought may represent the capacity of mind (as an ‘observing ego’) to engage in cognitive processing as *propagating events*. Because of the capacity of mind (as the energy pure mental) to keep ‘oscillating records’ (as previously described), there is a cause and effect relationship as relates to reactions. These records of actions and thus reactions act as ‘seeds’ that will eventually bear fruit by way of relationships [6] that manifest as cyclic though not repetitive embodiments. The ability of mind (as an ‘observing ego’) to think more complexly (i.e., with conceptual thoughts and memories) rather than simply non-conceptually (i.e., interoception, exteroceptive body, and proprioception) may be due to the strength of the Top-down mechanism (previously described). Because this mechanism determines the capacity of mind (as an ‘observing ego’) to ‘override’ oscillations of encoded change as the incoming signals sent by mind (as the energy pure mental). Accordingly, mind (as an ‘observing ego’) by way of its impulsive reactions and reactions with forethought engages in actions in response to primordial feelings (i.e., pleasant, unpleasant, or neutral) and FOKs that update the ‘oscillating record.’ Reactions in this context may then represent potential markers of conscious affective processing. The ‘intelligent’ behavior of mind evolves by way of reactions through a combination of autonomic (i.e., reflexive), and impulsive reactions as well as reactions with forethought rather than autonomic reactions only. In this model, ‘intelligent’ behavior is represented by a living being (e.g., invertebrate, mammal, bird, amphibian, reptile, or fish) as the ‘visual’ indicator of a unique embodied relationship (previously described). This character that emerges by way of consciousness thus will exhibit more or less ‘intelligent’ behaviors.

**Figure 5.**
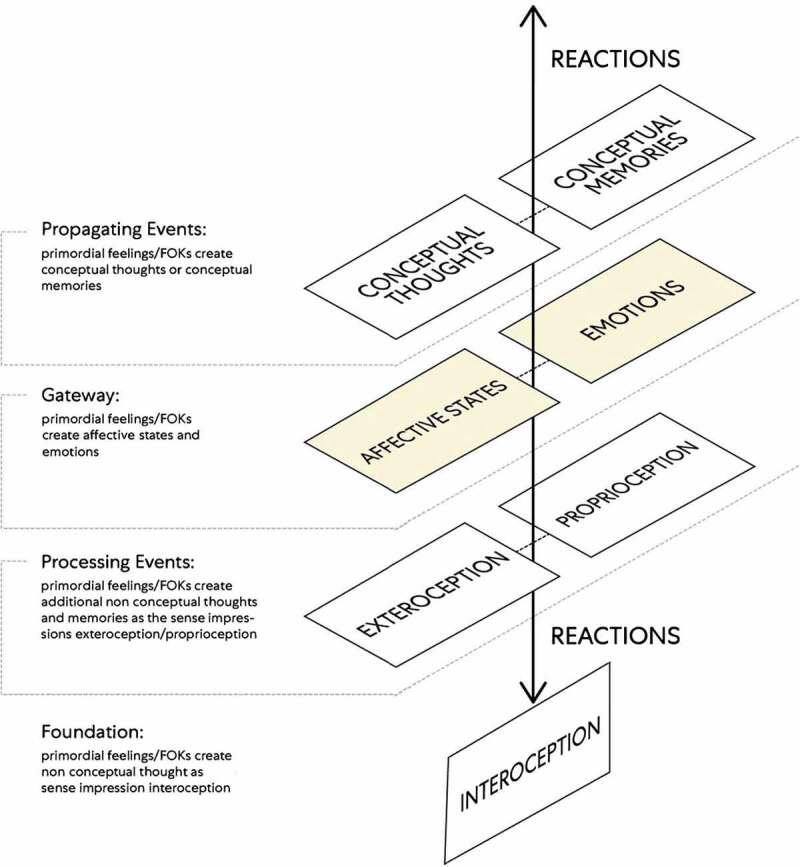
This components map model depicts the key role of interoception. The attention and reactions of mind (as an ‘observing ego’) are how it makes interoceptive predictions across a hierarchy of perceptual processing [3]

The relationship of mind as embodied energies (i.e., pure mental and mental images) and (as an ‘observing ego’) can be conceptualized by way of a components map model (see, Figure 5).

An illustration of how this model may be applied is in explaining the integration of both the Bottom-up and Top-down mechanisms (previously described) to conceptualize interoception’s relevance. In this model cognitive construction is layered whereby non-conceptual thought is represented by the lower layers (as the foundation and processing events), affective states, and emotions demarcate a layer that acts as a *gateway* to a layer of conceptual thoughts and memories (as propagating events). Accordingly, the non-conceptual sense impressions (i.e., interoception, exteroception, proprioception) of mind (as an ‘observing ego’) are due to its autonomic (i.e., reflexive) reactions depicted below the gateway (i.e., the affective states and emotions layer). Whereas the mental events (i.e., conceptual thoughts and conceptual memories) of mind (as an ‘observing ego’) are due to its impulsive reactions and reactions with forethought depicted above the gateway (i.e., the affective states and emotions layer).

Many animals display bodily and behavioral changes consistent with the occurrence of affective states similar to those seen in humans [25]. Accordingly, affective processes and their role in decision-making open up the possibility of studying choice behavior as a potential marker of animal affect and even conscious emotion [25]. Because the understanding of human characters is facilitated by their emotional reports; perhaps work in posing questions about conscious affect in animals starts with human models [see 25]. In humans, conscious processing involves information processing that can be deliberately controlled [25]. Human models thus can be used to identify candidate criteria for conscious emotion, which can be applied to observations of behavior in different animal species [25].

## Interoception, dysregulation, and interoceptive abnormalities

By using interoception to create its own internal maps, mind (as an ‘observing ego’) is thus informed to emotional experience and motivating regulatory behavior [15]. Accordingly, these internal maps shape its approach or avoidance tendencies [4] and perception of well being [29]. To the extent that mind (as an ‘observing ego’) is sensitive to interoceptive signals [4], such signals guide its decision-making [30]. However, sensitivity to interoceptive signals is not without its price [4] and thus when body sensation is irregular, mind (as an ‘observing ego’) experiences a wide range of emotional dysregulation and interoceptive abnormalities. Accordingly, interoceptive sensitivity of mind (as an ‘observing ego’) may either contribute to or detract from well-being [4]. In this way, the emotional experiences of mind (as an ‘observing ego’) arise from cognitively contextualized perception of changes in bodily states and beliefs about the causes of interoceptive signals [3]. When mind (as an ‘observing ego’) experiences abnormal interoceptive inference [3] through attention and autonomic reflexes this causes disorders in its emotional processing and interoceptive experience. Accordingly, there exist half-conscious or unconscious dysfunctional beliefs that drive its negative moods [2]. The mood and anxiety disorders of mind (as an ‘observing ego’) have been linked to failures to appropriately anticipate changes in interoceptive states [31]. This characteristic may jointly express a primary role of interoceptive states in anxious apprehension, sensitivity to a potential threat, and excessive avoidance of perceived harm that is a common underlying vulnerability to anxiety disorders [32]. Other conditions likely marked by interoceptive disturbances include drug addiction [33], depression, post-traumatic stress disorder, somatic symptom disorders [34], chronic pain [35], Tourette’s syndrome, and other tic disorders, borderline personality disorder [36], obsessive-compulsive disorder [36], autism spectrum disorder [37], and functional developmental disorders [38].

The interoceptive perception by mind (as an ‘observing ego’) is strongly shaped by expectations [14] and thus encoded signals it receives from mind (as the energy pure mental). Mind (as an ‘observing ego’) may sense interoception in ways that may be classified by these key distinctions [36]:

1. Painful or nonpainful
2. Occurring across a wide range of negative and positive valences and high/low arousal
3. Occurring (usually) outside of conscious awareness, except for pain sensations
4. Often but not always, experienced during instances of homeostatic perturbation

Accordingly, interoception is integral to the higher-order cognition of mind (as an ‘observing ego’). There is an association between low interoceptive sensitivity and alexithymia (a difficulty identifying one’s emotions [39];. Alternatively heightened interoception may relate to any kind of interoceptive experience. When related to cognitive aversion, it may be an unpleasant experience that is broadly characterized by a *not just right sense* that makes mind (as an ‘observing ego’) want to do something to neutralize it [40]. Anything it does in response to the thought “I must do this” that is effortful and has to be done in response to the not just right experience could very well be neutralizing behavior. Unpleasant primordial feelings and FOKs associated with bodily sensations (i.e., interoception) may be experienced by mind (as an ‘observing ego’) as being fundamentally distressing because they signal in an intense but vague way that things are not right [40]. This often triggers mental proliferation as a series of events [18], and interoception may be encoded into affective feelings. These include nociception, disgust, and empathy [19]. The strategy outlined in this model is where mind (as an ‘observing ego’) uses mindfulness-based interventions (MBIs) and interoceptive exposure (IE) activity to break up the automaticity of habitual reactions to primordial feelings and FOKs associated with interoception and do something different [40]. Accordingly, IE is a behavioral intervention that reduces anxiety sensitivity and distress associated with somatic sensations through interoceptive conditioning [41]. This involves confronting physical sensations that have become strongly associated with negative emotional experiences [41]. Although a full description of IE is beyond the scope of this article it has potential as a transdiagnostic intervention when interoceptive sensitivity is targeted [41]. Thereby “If you feel you are in a black hole, don’t give up. There’s a way out.” [42]. Exposure and response prevention (ERP) is the most efﬁcacious psychological treatment [43], and thus by way of IE the science of interoception is implemented as a transdiagnostic intervention strategy. Accordingly, the mind itself investigates the mind to address its intolerance of the physical sensations that signify an emotional state. Thereby it experientially dispels the fundamental delusions that generate so much suffering for itself and ‘others.’

MBIs such as attention regulation practice and insight orientated practice may maximize long-term learning by introducing ubiquitous real-world challenges that have the added beneﬁt of maximizing the retrieval of newly learned information [43]. An example of how this might be practiced [40]:

1. During multiple and/or sustained daily sessions of increasing duration, unpleasant interoception is the focus of attention and object of meditation to the exclusion of everything else.
2. Between meditative sessions unpleasant interoception is used as the object of awareness to anchor reactivity to unpleasant primordial feelings and FOKs.

Therefore, mind (as an ‘observing ego’) does not move away from the unpleasant interoceptive experience but instead stays with it and learns to not push the not right experience away but instead just let it be [40]. Through MBIs unpleasant interoception is used to focus the reactivity of mind (as an ‘observing ego’) to unpleasant primordial feelings and FOKs thus break up the automaticity of being driven by them. In this way, mind (as an ‘observing ego’) decides to approach these internal sensations through exposure to the interoceptive not just right sense instead of engaging in neutralizing activity [40].

## Conclusion

The novelty of this theory perhaps lies in its simplicity and transdisciplinary application which the author argues that any ‘theory of everything’ must do. This theory has merely supplied some details of this process through a reinterpretation of existing theories. One of the main goals in doing this is to bridge the gap between the way things really are and the way things seem to be. Accordingly, when oscillations of change are transformed by mind (as an ‘observing ego’) it experiences primordial feelings and FOKs that precede image-making and marks the first moment of subjectivity while thinking. A flow of cognition begins with primordial feelings and FOKs that are inseparable from a ‘sense of self’ as interoception [40]. The function of this might be to provide a metastable network that enables mind (as an ‘observing ego’) to compute the significance of a stimulus in its complex global context [7]. Due to interoception, mind (as an ‘observing ego’) has the capacity (to varying degrees) to determine if what it is observing matches its expectation of what it thinks it should be [3,7]. It will use the internal state of the body to determine if what it is feeling represents a state of affairs that is potentially threatening or rewarding [7].

Because mind (as the energy pure awareness) creates a substrate for embodied relationships there is a fundamental quality of mind that is present at all times: the quality of knowing, of being aware that without this cognitive quality, one cannot speak of mind or consciousness [10]. However, mind (as the energy pure awareness) exists without a characteristic or trait by which one can describe it by observation, measurement, or combination. This arguably is the key to understanding why each branch of science that emerges in consciousness has a ‘mystery to solve.’ The consciousness that mind (as an ‘observing ego’) creates for itself according to this theory describes innumerable problems and its attempts to solve them. This may be represented by Einstein’s famous equation E = mc^2^ which shows that energy and mass are interchangeable. In this equation, a squared quantity of energy can be converted into a particle of matter (and its mass m will be squared) [10]. The question becomes where does the conversion take place? The answer to this question is consciousness where energy relatively becomes matter. The Top-down mechanism and Bottom-up mechanism discussed in this article give rise to a “strange loop,” between mind (as the energy pure mental) and mind (as an ‘observing ego’). Where “the physical world gives rise to observers, who in turn conceive of the physical world in which they emerged.” (John Archibald Wheeler 1911–2008). It is by way of consciousness that mind (as an ‘observing ego’) attains knowledge, which may give it the capacity to perceive a very definite meaning to the whole mind-body-consciousness set up.

## Data Availability

Data sharing not applicable to this article as no datasets were generated or analyzed during the current study.
